# A novel 2 bp deletion variant in Ovine-*DRB1* gene is associated with increased Visna/maedi susceptibility in Turkish sheep

**DOI:** 10.1038/s41598-021-93864-8

**Published:** 2021-07-14

**Authors:** Yalçın Yaman, Veysel Bay, Ramazan Aymaz, Murat Keleş, Yasemin Öner, Eden Yitna Teferedegn, Cemal Ün

**Affiliations:** 1 Department of Biometry and Genetics, Sheep Breeding and Research Institute, 10200 Bandırma, Balıkesir Turkey; 2 Aegean Agricultural Research Institute, Menemen, İzmir Turkey; 3 Department of Biometry and Genetics, Agricultural Faculty, Bursa Uludag University, 16000 Bursa, Turkey; 4 Armauer Hansen Research Institute, Biotechnology, and Bioinformatics Directorate, Addis Ababa, Ethiopia; 5 Department of Biology, Faculty of Science, Aegean University, 35000 İzmir, Turkey

**Keywords:** Animal breeding, Genetic association study

## Abstract

Visna/maedi (VM) is a multisystemic lentivirus infection of sheep that affecting sheep industry across the globe. *TMEM154* gene has been identified to be a major VM-associated host gene, nevertheless, a recent study showed that the frequency of the VM-resistant *TMEM154* haplotypes was very low or absent in indigenous sheep. Thus, the present study was designed to determine other possible co-receptors associated with VM. For this purpose, *DRB1* gene, which is renowned for its role in host immune response against various diseases was targeted. A total number of 151 case–control matched pairs were constructed from 2266 serologically tested sheep. A broad range of *DRB1* haplotype diversity was detected by sequence-based genotyping. Moreover, a novel 2 bp deletion (*del*) in the *DRB1* intron 1 was identified. For the final statistic, the sheep carrying VM-resistant *TMEM154* diplotypes were removed and a McNemar’s test with a matched pairs experimental design was conducted. Consequently, it was identified for the first time that the 2 bp *del* variant is a genetic risk factor for VM (*p* value 0.002; chi-square 8.31; odds ratio 2.9; statistical power 0.90) in the dominant model. Thus, negative selection for 2 bp *del* variant could decrease VM infection risk in Turkish sheep.

## Introduction

Small ruminant lentiviruses (SRLVs) such as caprine arthritis encephalitis virus (CAEV) in goats and Visna/maedi virus (VMV) in sheep cause prevalent chronic infections across the globe. The infection results in multisystemic inflammation like arthritis, mastitis, lymphadonopathy, interstitial pneumonia, and meningoencephalitis. SRLV infections are mainly characterized by an insidious onset, slow disease progression, and ultimate fatality. Both viruses (CAEV and VMV) are similar in their genetic structure that paves the way for the cross-species infection transmission between sheep and goat^1–3^.

Visna/maedi (VM) has been reported in Europe^4,5^, North America^6^, South America^7^, Asia^8^, and Africa^9,10^. Only New Zealand and Australia are yet VM free, nonetheless, these countries are suffering from CAEV^11^. VM was first reported in Turkey in 1987^12^. Regardless of sampling methods, successive reports showed that VM seroprevalence was from 2.7 to 77.9%^13–15^. In a recent serosurvey on randomly sampled seven native Turkish breeds and four crossbreds (*n* = 2266), it was demonstrated that the breed-level mean seroprevalence was 12.7% ranging from 2 to 83.1%. In the same study, 13 out of 16 flocks were seropositive, and the flock level prevalence was 81.3%^16^.

There is no effective vaccine against VM as of the present. Once the animal acquires the VM virus, it will remain as a life long reservoir of the virus due to the persistency of infection^1^. Several studies reported indirect production losses caused by VM through low conception rates, reduced milk production, lower birth or weaning weight of lambs given by infected ewes^17^. On the other side, death or premature culling of infected animals are direct production losses^18^. The estimated VM associated production loss was up to 20% in the USA^19^ and 40% in UK^17^.

Testing and culling VM positive ewes and their lambs, the requirement of extra facilities to keep positive sheep until culling, and restocking ewes make the infection eradication efforts much more difficult in terms of cost and labor^20^. Moreover, screening and culling eradication strategy for VM does not guarantee long-lasting infection-free flocks as flocks will remain open to a new infection at any time. A typical example of such case is a field trial in 1979, where thirteen VM affected commercial flocks with 17% mean seroprevalence were subjected to screening and culling program for every 6 months. After two years, at the 5, 6, and 7th screening, all flocks were found seronegative. However, at the 8th test, three VM seropositive sheep were found where the exact source of the infection remained unclear^21^.

The search for ways to implement effective selective breeding strategies against lethal and chronic diseases like VM has been gaining increased attention worldwide. Several candidate loci such as ovar*-DRB1*^22,23^, *CCR5*^24^, *DPPA2/DPPA4* and *SYTL3*^25^, *ZNF389*^26^, and *TLR9*^27^ were reported to be associated with the VM serostatus and/or VM virus proviral load. Moreover, a case–control matched pairs experimental design revealed two haplotypes (haplotypes 1 and 4) in Exon 2 region of *TMEM154* gene having a major effect according to the recessive model on reducing susceptibility to VM in North American sheep^28^. Subsequent studies have confirmed this association in North American^29,30^, German, Iranian^31,32^, and Turkish sheep^16^. The frequencies of the protective *TMEM154* haplotypes, however, were reported to be significantly low or absent in some indigenous Turkish and Iranian sheep^16,32^. *TMEM154* encodes a transmembrane protein highly expressed in B lymphocytes. There are evidence that *TMEM154* variants associated with type 2 diabetes susceptibility in human^33,34^. Despite the effect of *TMEM154* on VM occurrence has been repeatedly reported, its biological role in the host response pathway against VM is not yet known. Although *TMEM154* has been identified to be a major gene regarding resistance/susceptibility to VM, there could be other possible co-receptors that affect VM occurrence. In case the possible co-receptors are detected; they could be used in selective breeding for VM in indigenous sheep breeds.

Earlier attempts by our team to investigate the association between some previously reported VM associated genes, i.e., *CCR5*, *ZNF389, TLR9* and VM serostatus did not reveal any significant result when a case–control experimental design was implemented (unpublished data). Here we aimed to investigate the possible link between Ovar-*DRB1* and VM serostatus. Ovar-*DRB1* was reported to be associated with VM in two independent studies^22,23^. *DRB1* is a MHC class II gene that encodes antigen-presenting receptor glycoproteins called histocompatibility molecules and plays a crucial role in recognizing peptides of pathogens and presenting them to the T-lymphocytes that eventually triggers host immune response. Because of its function in the immune system, *DRB1* gene has been associated with various diseases in sheep as well as in other mammalians (reviewed in^35^).

In the present study, a case–control matched pairs experiment was carried out to investigate the possible association between Ovar-*DRB1* variants and serostatus of VM in Turkish sheep. Furthermore, we have performed computational functional analysis to predict peptide–protein affinity and the possible effect of amino acid substitutions.

## Results

**Figure 1:**
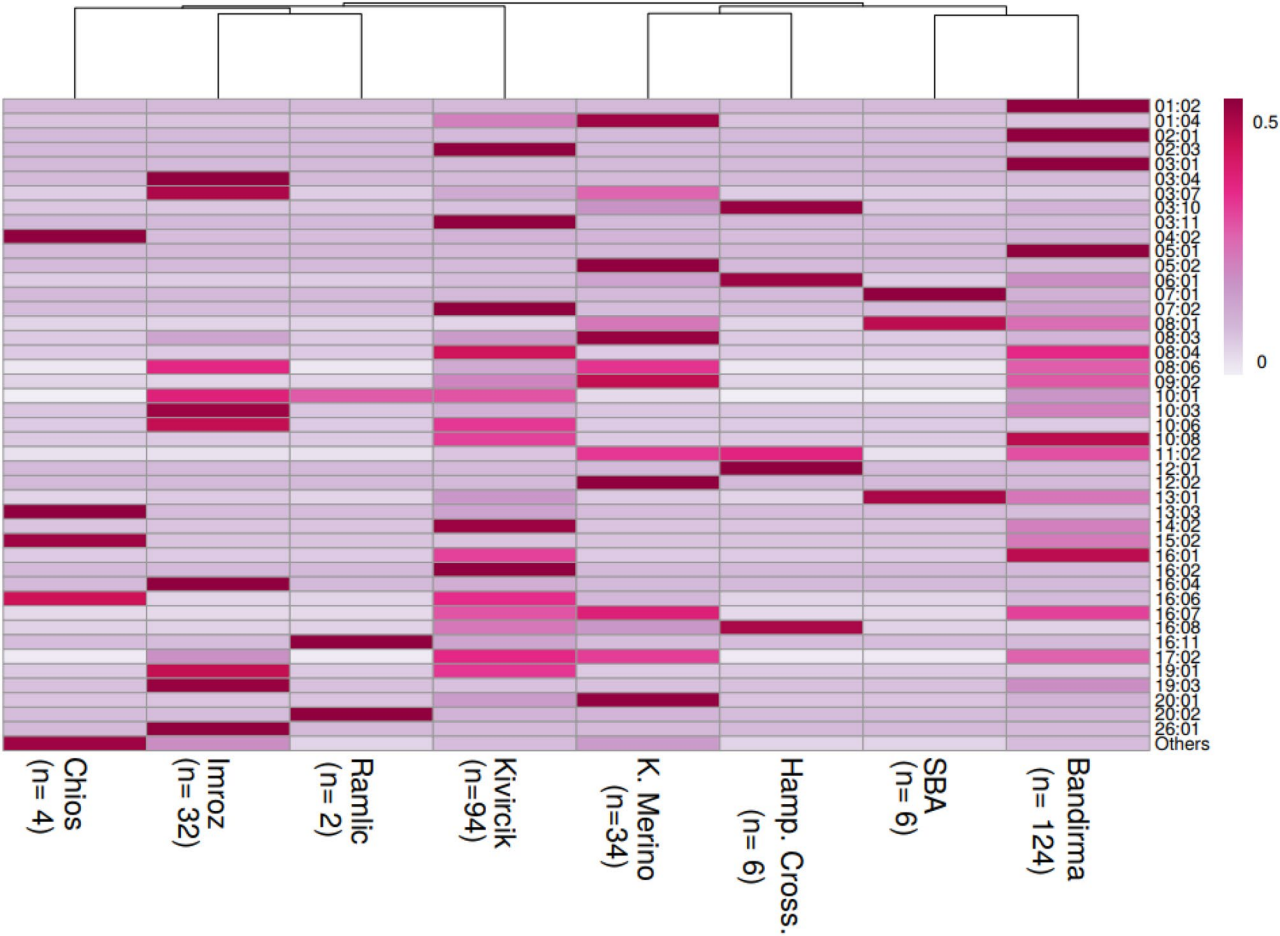
Distribution of *DRB1* haplotypes according to breeds.

**Figure 2:**
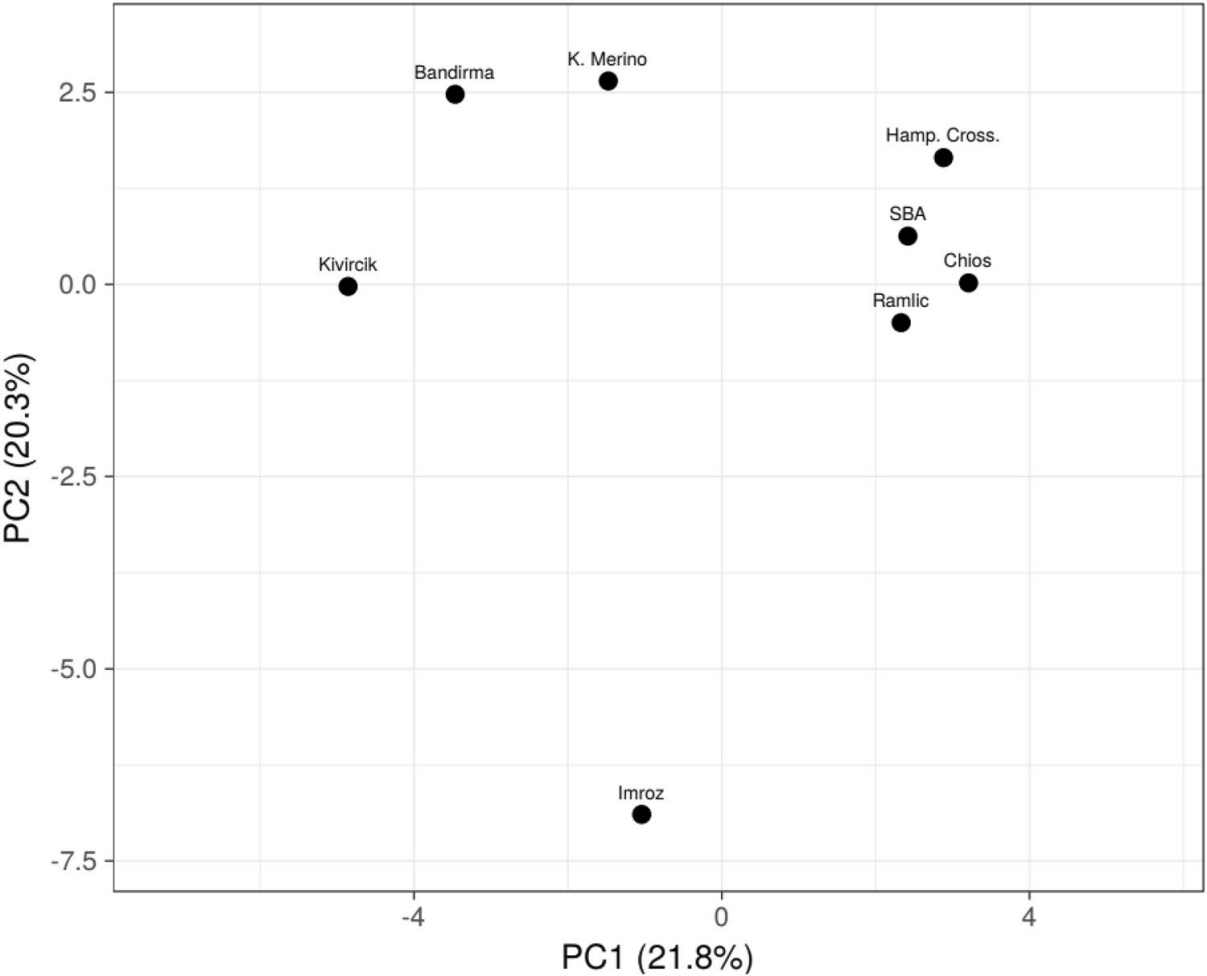
Principal components plot of *DRB1* haplotypes according to breeds.

Sequences obtained by using standard and universal M13 tagged primers showed 100% concordance. A total number of sixty-five *DRB1* Exon 2 haplotypes were estimated across all breeds. 10:01, 13:01, 09:02, and 08:01 were the most prevalent haplotypes which occurred at an overall frequency greater than 0.05, i.e., 0.19, 0.12, 0.09, and 0.09, respectively. Some of the haplotypes were breed-specific, whereas some haplotypes (*n* = 21) found as single copy across all sample set. Regarding the haplotypes which were detected at least two times across all breeds, broad haplotype diversity was observed in Kivircik (31), Bandirma (29), Merino (20), and Imroz (13; Fig. 1; Supplemental table S1). The principal components (PC) plot for PC1 (21.8%) versus PC2 (20.3%) demonstrated that SBA crosses, Hampshire crosses, Ramlic and native Chios were slightly clustered regarding *DRB1* genotypes according to breeds and the most distinct breed was Imroz (Fig. 2).

**Figure 3:**
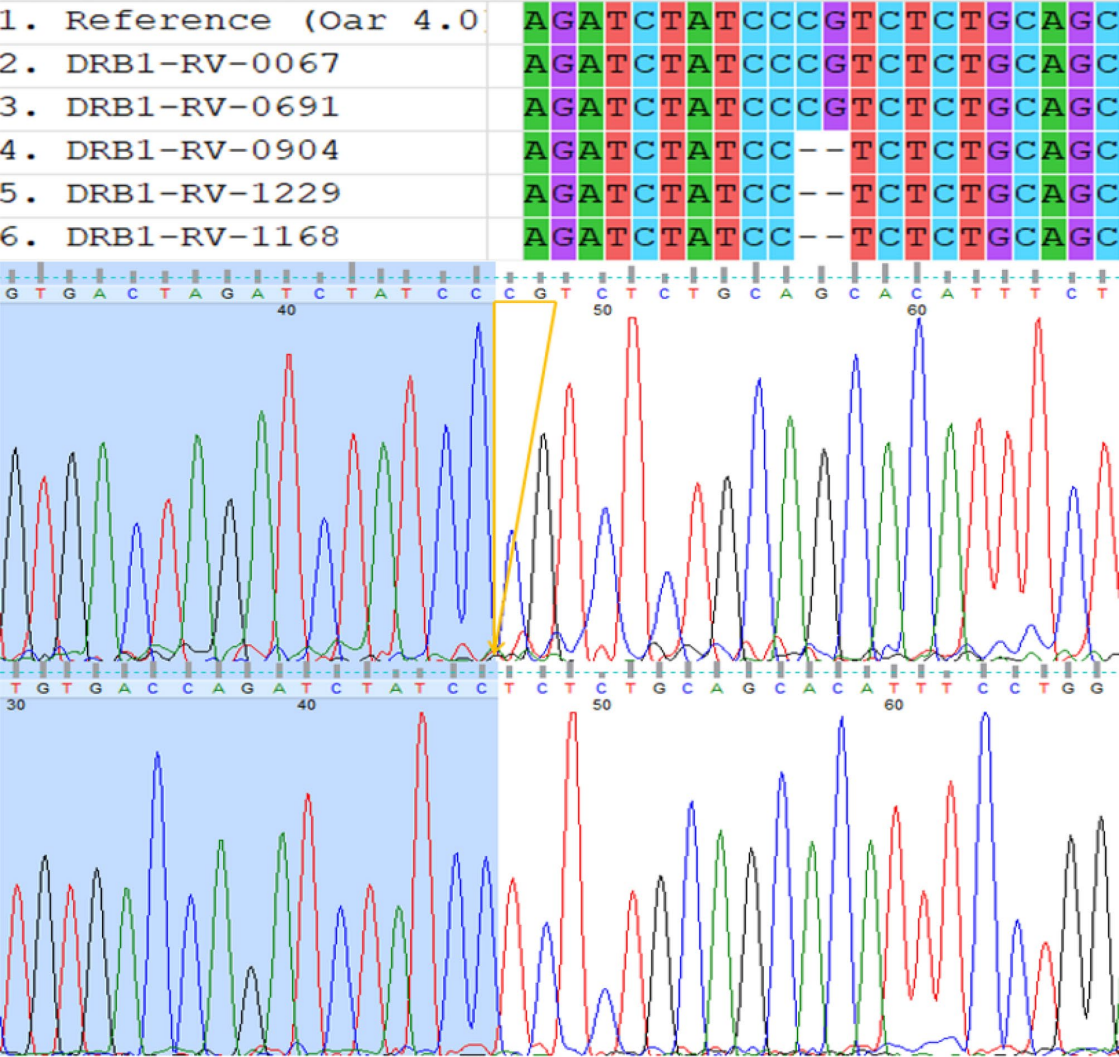
Alignment of *DRB1* haplotypes and physical position of 2 bp *del* variant. Last three sequence in the alignment and the bottom figure in the chromatograms are deleted variants.

**Figure 4:**
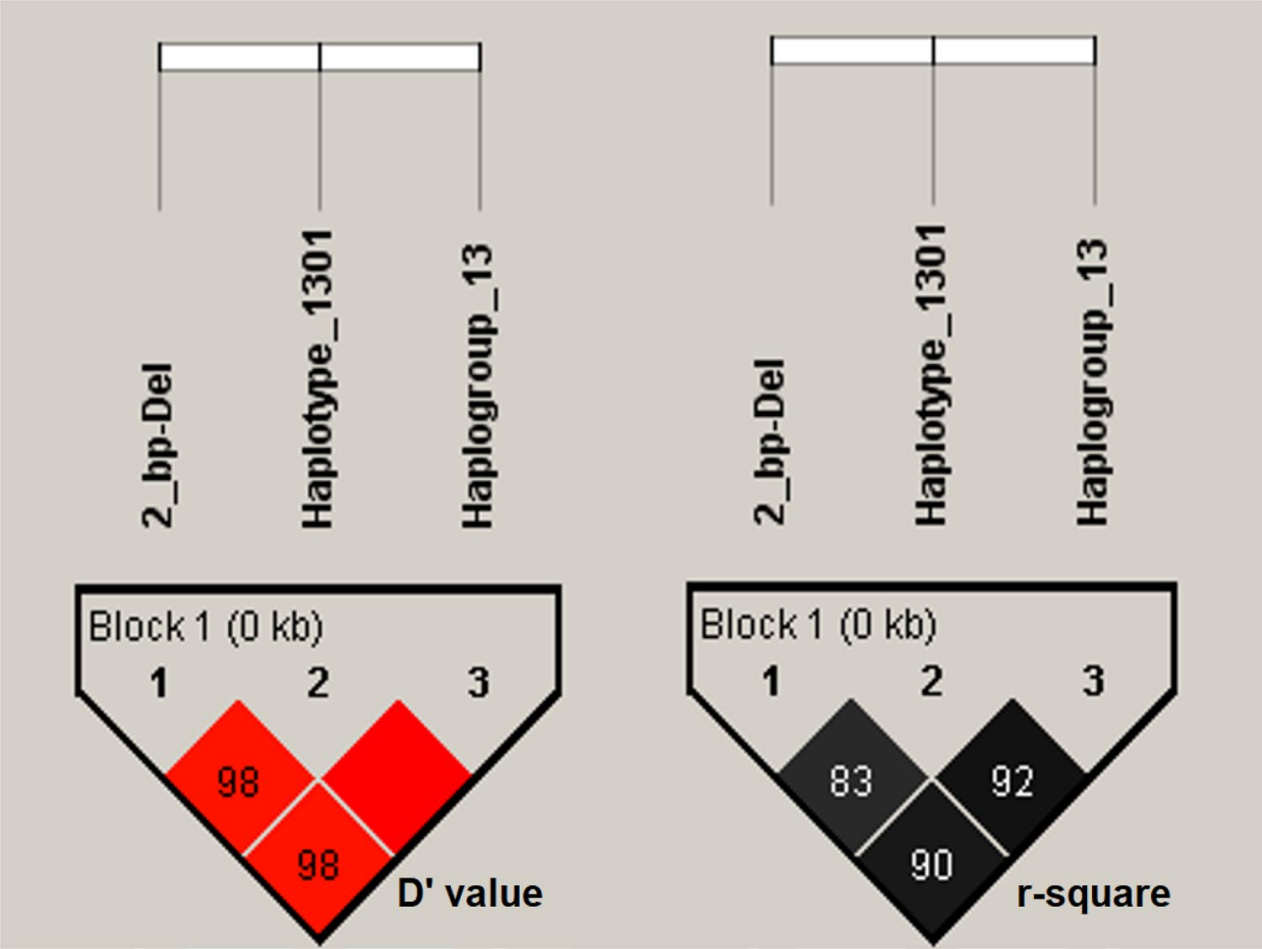
Linkage disequilibrium between 2 bp *del* variant, haplotype 13:01, and haplogroup 13.

Alongside *DRB1* Exon 2 haplotypes, a 2 bp deletion mutation (2 bp *del*) was identified at the Intron1 region of *DRB1* gene, just 11 bp upstream of Exon 2 (Fig. 3). Deleted nucleotides are “CG” and the exact genomic position of the deletion mutation is 20:27300971-72 (NC_040271.1). Strong Linkage Disequilibrium (LD) was observed between the 2 bp *del* mutation and 13:01 haplotype (D’ value, 0.984; r-squared, 0.836), moreover, the highest LD was identified between the 2 bp *del* mutation and detected haplogroup 13 (haplotypes 13:01 and 13:03; D′ value, 0.984; r-squared, 0.903; Fig. 4). Haplotype 13:02 was not informative for LD analysis as it was detected as only one copy. The 2 bp *del* mutation was not found in Karacabey merino, Ramlic, and Hampshire crosses. The frequencies of the 2 bp *del* mutation in Chios, Imroz, Kivircik, Bandirma, and SBA was observed at a range of 0.03 (Imroz) to 0.42 (SBA; Table 1).

**Table 1:** Frequencies of the 2 bp *del* mutation in 151 matched pairs panel.

Breed	Situation	*n*	2 bp *del*
Chios	Native	4	0.250
Imroz	Native	32	0.031
Kivircik	Native	94	0.144
Merino	İmproved breed	34	–
Ramlic	İmproved breed	2	–
SBA	Research flock	6	0.417
Hampshire crosses	Research flock	6	–
Bandirma	Research flock	124	0.181
	Overall	302	0.134

A McNemar’s test was performed over 142 matched pairs to investigate the correlation between the 2 bp *del* mutation or the most prevalent haplotypes (10:01, 13:01, 09:02, and 08:01) and VM serostatus (Supplemental table S2). Both recessive and dominant models were tested. In McNemar’s test, the sum of the discordant pairs (1;0 and 0;1) is expected to be higher than 25 for statistical significance. Accordingly, all of the tested variants were observed to have higher than 25 discordant pairs for the dominant model but not for the recessive model. A statistically significant correlation was observed between VM serostatus and haplotype 13:01 and the 2 bp *del* mutation according to the dominant model (Table 2), among these two variants, however, the most significant association was detected between the 2 bp *del* mutation and VM serostatus (exact *p* value, 0.005; chi-square, 6.89; odds ratio, 2.36; CI 95) upon computing the dominant model. Statistical power analysis was performed using real sample size (142 matched pairs) and percentage of discordant pairs (33%). Hence, the statistical power of the first analysis was calculated to be 0.87 (*p* < 0.05).

**Table 2:** McNemar’s statistics of common *DRB1* haplotypes and 2 bp *del* mutation.

Estimated haplotypes	Model^a^	*n*^*b*^	McNemar quadrants				*b* + *c*^*c*^	*n*	(*b* + *c*)/*n*	OR CI_95_			*χ*^2^	*p* value (exact)
			1;1	1;0	0;1	0;0								
			*a*	*b*	*c*	*d*				OR	Lower	Upper		
13:01	1 copy	58	11	23	13	95	36	142	0.25	1.77	0.90	3.5	2.25	0.067
13:01	1 or 2 two copy	62	11	14	26	91	40	142	0.28	1.86	1.00	3.6	3.03	0.042
13:01	2 copy	4	0	3	1	138	4	142	0.03	3.00	0.30	28.8	0.25	0.5
**Haplogroup 13 (13:01, 13:02, and 13:03)**														
H/Y61T^6^	1 copy	62	11	27	13	91	40	142	0.28	2.07	1.10	4.0	4.23	0.022
H/Y61T	1 or 2 two copy	66	11	30	14	87	44	142	0.31	2.14	1.10	4.0	5.11	0.013
H/Y61T	2 copy	4	0	3	1	138	4	142	0.03	3.00	0.30	28.8	1.00	0.5
2 bp *del*	1 copy	64	10	31	13	88	44	142	0.31	2.39	1.20	4.6	6.57	0.006
2 bp *del*	1 or 2 two copy	69	11	33	14	84	47	142	0.33	2.36	1.30	4.4	6.89	0.005
2 bp *del*	2 copy	5	0	3	2	137	5	142	0.04	1.50	0.30	0.9	0.00	0.625
**Lacking *TMEM154* protective diplotypes**														
2 bp *del*	1 copy	43	4	26	9	53	35	92	0.38	2.89	1.40	6.2	7.31	0.004
2 bp *del*	1 or 2 two copy	47	4	29	10	49	39	92	0.42	2.90	1.40	6.0	8.31	0.002
2 bp *del*	2 copy	4	0	3	1	88	4	92	0.04	3.00	0.30	28.8	0.25	0.5

Table abbreviations: *a,* indicate the model that is “at least one copy” is dominant and “two copy” is recessive model; *b*, total number of ewes that are carriers of interested haplotype as one and/or two copies; *c*, sum of discordant pairs; OR, Odds ratio; χ^2^, the McNemar’s test statistic with continuity correction; *d*, representing haplogroup 13 (*1301 and *1303).

Alongside to strong LD between the 2 bp *del* and haplotype 13:01, it was identified that Histidine (H) or Tyrosine (Y) substitution to Threonine (T) at codon 61 (H/Y61T) was also linked to both 2 bp *del* and 1301 haplotype (Fig. 4). Among 124 ovine *DRB1* haplotypes deposited in Immuno Polymorphism Database (IPD), the 61T amino acid variant is specific to 13:01, 13:02, 13:03, 05:01:01, 05:02:01, 05.03:01, 26:01, and 29:01 haplotypes of which only 13:01, 13:02, 13:03, and 26:01 were detected in the present study with the overall frequencies of 0.12, 0.003, 0.007, and 0.005, respectively. Another LD analysis was performed over the 2 bp *del* mutation and the haplogroup 13 (haplotypes 13:01, 13;02, and 13:03) which are carrier of 61T missense variants. Since haplotype 26:01 was detected as three copy across all breeds and neither those were not carrier of 2 bp *del* variant, this haplotype was excluded from this analysis. Eventually, almost a perfect LD was observed between the 2 bp *del* variant and the 61T amino acid carrier haplogroup 13 (D′ value, 0.985; r-squared ≥ 0.903; Fig. 4). Thus, second McNemars’s test for the haplogroup 13 with 44 discordant pairs revealed a similar association with the 2 bp *del* mutation (exact *p* value, 0.013; odds ratio, 2.14) in dominant model (Table 2).

Our case–control matched pairs panel was also available for *TMEM154* genotypes which were identified to have a major effect for the recessive model on VM resistance/susceptibility^16,28–32^. Finally, to test the relative protection of the wild type *DRB1* genotype compared to the 2 bp *del* variant in the absence of the protective effect of *TMEM154*, these matched pairs were sorted and analyzed again. Briefly, if at least one member of a pair is a carrier of protective *TMEM154* diplotypes (1;1, 1;4, or 4;4), these pairs were removed from the data set, thus, 92 matched pairs lacking the protective *TMEM154* diplotypes remained (Supplemental table S3). Consequently, the third McNemar’s test for correlated proportion was performed on this data set with 39 discordant pairs (1;0 and 0;1). It was determined that 2 bp *del* mutation was still significantly associated with increased susceptibility, and the wild type ones were associated with relative resistance to VM despite lacking the protective *TMEM154* diplotypes (exact *p* value*,* 0.002; chi-square, 8.31; odds ratio, 2.9; CI 95). The statistical power of this analyzis was calculated to be 0.90 (odds ratio, 2.9; CI 95; *p* < 0.05). According to our results, the 2 bp *del* mutation in *DRB1* Intron1 was identified as a genetic risk factor in dominant model, i.e., having one or two copies of 2 bp *del* mutation was found to increase the risk of contracting the VM virus by 2.9 fold (Table 2).

In silico peptide–protein docking analysis was performed to predict the docking affinity between VM virus Gag protein antigenic epitope and *DRB1* high-frequency haplotypes i.e.13:01, 10:01, 08:01, and 09:02. The prediction result revealed docking scores were variable among these four haplotypes and both of the two tools (HDOCK and HEPDOCK) computed relatively lower docking score for VM associated haplotype 13:01 when compared to other common haplotypes (Table 3). Additionally, functional analysis of point mutation effect was predicted for H/Y61T substitutions using two popular web applications. Accordingly, PROVEAN predicted the H61T and Y61T substitutions have “deleterious” effect while, PANTHER predicted the same substitutions to exhibit “probably benign” effect (Table 4).

**Table 3:** Epitope-protein (DRB1) docking for VM virus Gag protein.

Epitope	Epitope Id	Antigen; VM Virus	Detected haplotypes with > 0.05 frequency	HDOCK	HEPDOCK
				Docking score	Docking score
GNRAQKELIQGKLNEEAERWVRQNPPGPN	21507	Gag protein	13:01	− 190.49	− 197.160
			08:01	− 197.28	− 211.041
			10:01	− 203.94	− 235.318
			09:02	− 205.90	− 201.308

**Table 4:** Point mutation effect prediction of the (H/Y) to (T) amino acid replacement at codon position 61.

Amino acid substitution	Used web tools	Predicted effect	Predicting score/cutoff	Score
H61T	PROVEAN	Deleterious	− 2.5	− 5.219
	PANTHER	Probably benign		0.02
Y61T	PROVEAN	Deleterious	− 2.5	− 7.300
	PANTHER	Probably benign		0.02

PROVEAN: Variants with a score equal to or below − 2.5 are considered “deleterious”, variants with a score above − 2.5 are considered “neutral”.

## Discussion

As in many other mammalians, there are several identified ovine *DRB1* gene variants. According to IPD records, 124 ovine *DRB1* alleles or subtypes have been deposited so far. In the present study, a broad genetic diversity in Exon 2 region of *DRB1* gene was identified in Turkish sheep. There were 44 different haplotypes detected in at least two times (Supplemental table S1). The highest relative allelic diversity (the number of the detected different alleles over total alleles in each breeds) was in Chios breed (0.625) and the lowest in Kivircik breed (0.176).

Exon 2 region of the ovar *DRB1* gene has gained great attention of researchers having a broad genetic diversity and its key role in immune defense. Previously, the association between various *DRB1* alleles and different diseases such as Cystic Echinococcosis^36^, faecal egg count of gastrointestinal nematodes^37,38^ have been reported. The association between *DRB1* gene and resistance/susceptibility to VM have also been reported in two different studies. In the first study, it was found that *DRB1* haplotypes 04:03 and 07:12 were significantly associated with lower provirus levels of the ovine progressive pneumonia^22^, which is the counterpart of VM in North America, and in the second study, it was reported that the haplotype 03:25 was associated with the susceptibility to VM^23^. However, these reported haplotypes were not detected in the present study.

According to present findings, strong LD was detected between 2 bp *del* variant and 61T amino acid substitution which was found in 13:01 and 13:03 haplotypes in our genotype panel. To further investigate, fifty Ovar-*DRB1* sequences consisting of 2 bp *del* variant in intron 1 were obtained from GenBank (*Accession numbers:* MG000511.1, MG000512.1, MG000515.1, MG000516.1, MG000518.1, MG000538.1 to 552.1, and MG000555.1 to 586.1). All the sequences belonged to the Djallonke and Sahelian native sheep breeds of Ghana in West Africa. These sequences were searched using BLAST on IPD server, and the results revealed that all of these sequences were compatible with the haplotype 13:02 (D′ value, 1; r-squared, 1). Archaeological evidence and retrovirus integration analyses suggest that selection for desired traits common to modern sheep first began in Fertile Crescent including the Anatolia region of Turkey, and spread to Africa, Europa, and other parts of Asia^39^. All the native breeds in the present study were carriers of the 2 bp *del* mutation, but improved breeds by backcrossing (Karacabey merino and Ramlic) were not. Other two research flocks, Bandirma which was bred from native Kivircik breed and SBA crosses, were also carriers of this mutation. When the GenBank records and our findings evaluated together; it can be inferred that having been found in African and Turkish native breeds, 2 bp deletion is an ancient mutation and perfectly linked to both haplogroup 13 (13:01, 13:02, and 13:03) and 61T amino acid variation. Furthermore, it could be speculated that 61T amino acid substitution, either alone or together with other codons, may be the causative variant for increased susceptibility against VM infection. However, case control studies are required with other 61T carrier haplotypes i.e. 05:01:01, 05:02:01, 05.03:01, 26:01, and 29:01 to strengthen this hypothesis. Furthermore, the LD status between 2 bp *del* and 61T amino acid variant on these haplotypes remained ambiguous. Despite these limitations, it was clearly demonstrated that 2 bp *del* mutation in the intron 1 of the ovine *DRB1* is a strong predictor for the 61T amino acid variant in haplogroup 13, and significantly associated with increased susceptibility against VM.

In a recent study, the VM resistant *TMEM154* haplotypes in Turkish native sheep breeds were detected either at a very low frequency or complately absent (0 to 0.12) when compared to the improved breeds by backcrossing^16^. A similar observation was made in Iranian native sheep breeds^32^. Hence, selective breeding regarding protective *TMEM154* genotypes might be a long lasting process to improve herd level genetic resistance to VM for native breeds. Alternatively, introgression or gene editing technology, i.e., CRISPR might be required for the native breeds lacking resistant *TMEM154* haplotypes. Otherwise, 2 bp *del* mutation and linked H/Y61T amino acid substitution was found a range of 3–25% indicating that negative selection for 2 bp *del* mutation could provide a chance for relatively rapid genetic improvement against VM disease for some native sheep.

From the first matched pairs panel, 21 ewes were carriers of susceptible *DRB1* 2 bp *del* variant and protective *TMEM154* diplotypes (1;1, 1;4, or 4;4) at the same time. According to the previous reports, *TMEM154* protective diplotypes provide approximately threefold protection to VM, thus, it is expected that only 1/4 of these 21 ewes could be VM positive. Ten of these ewes, however, were VM positive which might indicate the 2 bp *del* variants potentially limits the protective effect of the *TMEM154.* Thus, further genetic improvement could be achieved using combination of these two genetic markers (*DRB1* and *TMEM154*) for selective breeding of both native and improved sheep breeds.

Genotyping of MHC II locus, including *DRB* genes is highly problematic due to the excessive variation in a relatively short exon often causes sequence reading errors. Therefore, bidirectional sequencing is needed to reduce bias. In fact, bidirectional sequencing does not guarantee error-free genotyping, therefore, it is highly recommended to clone sequences for increased precision while genotyping. However, detection of 2 bp *del* variation could be much easier and cost effective with availability of genotyping techniques such as Allele Specific PCR (AS-PCR), Restriction Fragment Length Polymorphism (RFLP), Single Strand Conformationel Polymorphism (SSCP), or Real-Time PCR. Besides, genotyping of 2 bp *del* variant using such techniques might result in much fewer genotyping errors when compared to the exon sequencing.

Identifying peptides of antigen is a key step in the development of therapeutic options for many chronic infectious diseases^40^. Predicting viral epitope especially is cost effective and time saving^41^. Despite bioinformatics prediction does not mimics experimental results, predicting epitopes plays important roles. The identified Gag protein antigenic epitope was relatively weakly bind a 2 bp *del* associated *DRB1* receptor allele i.e. 13:01 compared to the other high-frequency alleles i.e.10:01, 08:01 and 09:02. The variation in binding affinity and peptide core could be due to the highly polymorphic nature of *DRB1* especially the specific binding cores^42^. This finding possibly strengthen the susceptibility feature of 13:01 because of the poorly docking affinity between the antigenic epitope of VM virus and this haplotype which potentially interferes the immune response upon infection.

As observed in this study, H/Y61T substitution was strongly associated with 13 haplogroup. We hypothesized that the variant may maneuver the receptor core affinity to the viral antigenic epitope. This assumption is supported by the proclivity of peptides to bind to *DRB1* of a particular allele in human^43^. Similarly, variants of peptides could define ligand specific binding capacity^44^ which in turn is defined by the docking energy score. Further to that, according to PROVEAN web server result, the new variant (T) was predicted to have deleterious effect than the commonly observed variants at position 61. The results obtained from PANTHER and PROVEAN servers were slightly inconsistent. This could be due to the assumptions that the databases are based on. PROVEAN takes the 75% sequence homology modeling to predict the effect of amino acid substitutions while PANTHER is based on the evolutionary history of a domain assuming a preserved region has functional importance. Additionally, PANTHER predicts the function of a query against the already existing 100 species in the databases which does not include *Ovis aries*. The sum of the results in terms of docking affinity and predictive effect of point mutation could give us a clue on how different alleles and variants that are harbored in the haplotypes affect the cellular immune reaction in fighting against the viral invasion.

In conclusion, this is a pioneering study in the identification of the association between the 2 bp *del* variation in ovine *DRB1* intron 1 and VM serostatus in the presence and/or absence of the protective effect of the major gene *TMEM154.* The protective haplotypes of *TMEM154* were previously detected at a high frequency in improved breeds such as Karacabey merino and Ramlic, and for native breeds they were in very low frequencies or absent. Conversely, 2 bp *del* variant of the *DRB1* gene having a strong association with increased susceptibility to VM was found in many native Turkish breeds. Thus, considering the prevalence of VM in some native breeds, it is highly reasonable to take the *DRB1* 2 bp *del* variant into account for selective breeding to obtain infection-free sheep flocks.

## Methods

### Animals

In this study, three indigenous Turkish breeds (Chios, Imroz, and Kivircik), two improved breeds (Karacabey Merino and Ramlic), and three composite breeds (Blackhead merino crosses-SBA, Hampshire crosses, and Bandirma) were considered. Chios, Imroz, and Kivircik are ancient breeds that are well-adapted to their environment for thousands of years. Karacabey merino was improved by backcrossing in the 1940s in Sheep Breeding and Research Institute (SRI), whereas Ramlic was improved in the 1960s by the Turkish Ministry of Agriculture and Forestry. Both breeds have been closed for backcrossing for more than 30 years. The composite breeds: SBA, Hampshire, and Bandirma have been reared as research flocks at SRI. Ewes for the study were selected according to their serostatus, age, breed type, and flock from the 2017 serosurvey cohort of 2266 ewes of eleven flocks at six different locations^16^. To allow sufficient VM virus exposure and subsequent seroconversion, only the ewes that were two years or older ages were included (ages ranging from two to eight). Thus, a total number of 302 ewes (151 cases and 151 controls) were matched. Haplotype phasing and LD analysis were conducted over these matched pairs. All methods were performed in accordance with the relevant guidelines and regulations.

### Serological analysis

To determine the VM virus-specific antibody titer in the serum samples, an Enzyme-Linked Immunosorbent Assay (ELISA) was carried out. Briefly, serums were separated from fresh whole blood samples by centrifugation and were tested with Idexx CAEV/VMV total Ab ELISA commercial kit following the manufacturer’s manual. At the final step, ELISA plates were read at 450 nm wavelength by an ELISA plate reader. The sensitivity and specificity of the used ELISA kit have reported being 84.3% and 99.6%, respectively^45^.

### Genetic analysis

Genomic DNA was extracted from peripheral whole blood with EDTA using commercial spin-column DNA extraction kits according to the manufacturer’s instructions. Polymerase chain reaction (PCR) was carried out to amplify Exon 2 region of Ovar-*DRB1* gene using previously designed primers (DRB1_330_F: ATTAGCCTCYCCCCAGGAGKC and DRB1_329_R: CACCCCCGCGCTCACCTCGCCGC)^46^. Amplified products overlap the last 54 bp of *DRB1* Intron1 and 270 bp of the full sequence of Exon 2. Standard Sanger sequencing analysis was performed as follows: pre-purification of PCR products by Exo-SAP incubation followed by chain termination reaction with BigDye™ Terminator v3. 1 kit, post-purification with ethanol precipitation protocol, and capillary electrophoresis on AB 3500 genetic analyzer were performed sequentially. All samples were sequenced bidirectionally. Approximately 1/4 (28%) from all samples were selected randomly and amplified separately using the same primers with universal M13 primer tags (M13_F: GTAAAACGACGGCCAGT and M13_R: AACAGCTATGACCATG) and sequenced bidirectionally to validate the obtained sequences and check primers binding sites.

### Statistical analysis

Chromatograms were visualized using FinchTV v1.4.0 software (Geospiza, Inc) and aligned by MEGA v6.0 software^47^. *DRB1* Exon 2 haplotypes were estimated using Phase v2.1 algorithm^48^ and identified by Basic Local Alignment Search Tool (BLAST) in both National Center for Biotechnology (NCBI; https://www.ncbi.nlm.nih.gov/) and Immuno Polymorphism Database (IPD; https://www.ebi.ac.uk/ipd/). LD analysis was performed using Haploview v4.2 20^49^.

Host genotype and disease resistance/susceptibility association study requires the control of confounding factors such as variations in exposure intensity, exposure duration and other environmental factors such as climate, herd management, herd density, access to the pasture. Furthermore, it is required to increase statistical power to reduce false positive results potentially caused by the population stratification phenomenon. Therefore, association analysis was performed according to case–control matched pairs experimental design. A total of 151 matched pairs (151 cases and 151 controls) were constructed according to age, breed type, and flock. Briefly, a seropositive ewe was randomly matched with a seronegative one from the same flock, same breed type, and same age group. This matched pairs panel was used for haplotype phasing and LD analiysis. Due to limited animal numbers in SBA (6) and Hampshire crosses (6), Ramlic (2), and native Chios (4), these breeds were ignored, and statistical analysis was performed on the remaining 142 matched pairs (Table 5). The age information was not available for a small proportion of matched pairs panel (4.2%). Thus, these individuals were matched according to breed type and flock. The pairs were assigned to be (1;1), (1;0), (0;1), and (0;0) i.e. both case and control are carriers of genetic risk/protective factors (1;1), the case is the carrier of genetic risk/protective factors, but control is not (1;0), the case is not a carrier of genetic risk/protective factors, but control is (0;1), and both case and control are not carriers of genetic risk/protective factors (0;0). In this experimental design, the higher rate of the discordant pairs (1;0 and 0;1) provides higher statistical power. A McNemar’s test^50^ for correlated proportions was performed using these matched pairs. To determine the statistical power of the experimental design, a power analysis was conducted using the G*Power software^51^.

**Table 5:** Age and breed composition of the 142 matched pairs.

Age	Imroz	Kivircik	Karacabey Merino	Bandirma	Total	%
8	0	0	0	10	10	3.5
7	0	18	6	32	56	19.7
6	10	24	6	26	66	23.2
5	12	18	4	30	64	22.5
4	4	14	6	20	44	15.5
3	4	8	10	6	28	9.9
2	2	0	2	0	4	1.4
na	0	12	0	0	12	4.2
Total	32	94	34	124	284	100
%	11.3	33.1	12.0	43.7	100	

*na* not applicable.

### In silico analysis

The binding affinity of the *DRB* core to the peptide is a potential factor in presenting antigen to immune cells^52^. Identifying the viral epitope is the first step to calculate the affinity of the given antigenic peptide to the *DRB* cores. Gag protein epitope of VM virus was extracted from Immune Epitope Database and Analysis Resource (IEDB, http://www.immuneepitope.org). Gag protein epitope was used for further ligand–protein affinity analysis using HEPDOCK and HDOCK web servers^53^. Besides, the effect of the H/Y61T substitution which is specific to 2 bp *del* linked haplogroup 13 (13:01, 13:02, and 13:03) was predicted using PROVEAN (http://provean.jcvi.org/) and PANTHER (http://www.pantherdb.org) web applications.

### Ethics declarations

All animal procedures in the study were reviewed and approved by the local ethics committee of Sheep Breeding and Research Institute (Approval No. 1282412), and the authors complied with the ARRIVE guidelines.

## Supplementary Information

Supplementary Table S1.

Supplementary Table S2.

Supplementary Table S3.

Supplementary Legends.
