# Supplementary material for: A novel 2 bp deletion variant in Ovine-DRB1 gene is associated with increased Visna/maedi susceptibility in Turkish sheep
Source: Sci Rep. 2021 Jul 14;11:14435. doi: 10.1038/s41598-021-93864-8 (PMC8280167; doi:10.1038/s41598-021-93864-8)
Supplement: Supplementary file 4 — Supplementary Legends. [file 41598_2021_93864_MOESM4_ESM.docx]

**Additional information**

**Supplementary files**

**Table S1.  *DRB1* haplotype distributions according to breeds.**

Filename: DRB1 table S1_haplotype distribution.xlsx

**Table S2. McNemar’s case-control matched pairs panel.**

Filename: DRB1 table S2_McNemars case-control matched pairs.xlsx

**Table S3. McNemar’s case-control matched pairs panel lacking *TMEM154* protective diplotypes.**

Filename: DRB1 table S3_McNemars case-control matched pairs lacking TMEM154 protective diplotypes.xlsx
